# Challenging diagnosis of congenital malaria in non-endemic areas

**DOI:** 10.1186/s12936-018-2614-9

**Published:** 2018-12-14

**Authors:** Lorenza Romani, Stefania Pane, Carlo Severini, Michela Menegon, Gianluca Foglietta, Stefania Bernardi, Hyppolite K. Tchidjou, Andrea Onetti Muda, Paolo Palma, Lorenza Putignani

**Affiliations:** 1 Division of Immunology and Infectious Diseases, Research Unit in Congenital and Perinatal Infections, University-Hospital, Pediatric Department (DPUO), Bambino Gesù Children’s Hospital, Piazza Sant’Onofrio 4, 00165 Rome, Italy; 2 Unit of Parasitology, Bambino Gesù Children’s Hospital, Piazza Sant’Onofrio 4, 00165 Rome, Italy; 3 Istituto Superiore di Sanità (ISS), Viale Regina Elena 299, 00161 Rome, Italy; 4 Department of Laboratories, Bambino Gesù Children’s Hospital, Piazza Sant’Onofrio 4, 00165 Rome, Italy; 5 Unit of Human Microbiome, Bambino Gesù Children’s Hospital, Viale San Paolo 15, 00146 Rome, Italy

**Keywords:** Congenital malaria, HIV, Bicorial, biamniotic pregnancy, Malaria laboratory panel, *Plasmodium falciparum* genotyping

## Abstract

**Background:**

Congenital malaria is usually defined as the detection of asexual forms of *Plasmodium* spp. in a blood sample of a neonate during perinatal age if there is no possibility of postpartum infection by a mosquito bite. The incidence of congenital malaria is highly variable and seems related to several factors, such as different diagnostic methods for *Plasmodium* spp. detection, and area in which the epidemiologic analyses are performed. In non-endemic countries, cases of congenital malaria are rare. Hereby, a case of a congenital malaria in an HIV exposed child is reported.

**Case presentation:**

A 2-month-old male child was admitted to Bambino Gesù Children’s Hospital due to anaemia and exposure to HIV. He was born prematurely in Italy by cesarean section at 34 weeks’ gestation after a bicorial, biamniotic pregnancy by a migrant woman from Nigeria. He was the first of non-identical twins. Combined with anaemia, spleen and liver enlargement was noted, malaria was hypothesized. Malaria laboratory panel was performed on the newborn, mother and other twin blood samples, as follows: (i) malaria rapid diagnostic test (RDT); (ii) Giemsa-stained thick and thin blood smears for *Plasmodium* spp. identification and parasitaemia titration; (iii) molecular screening and typing of *Plasmodium* spp. by multiplex qualitative PCR assay based on 18S rRNA gene. Genotyping of *Plasmodium falciparum* isolates from mother and child was performed by neutral microsatellite and highly polymorphic marker amplification.

**Conclusions:**

The maternal RDT sample was negative, while the infant RDT was positive; in both cases microscopy of blood smears and PCR showed infection with *P. falciparum*. Two of the genotypic molecular markers displayed different allelic variants between the two samples. This difference could imply infection multiplicity of the mother during the pregnancy, possibly harbouring more than one isolate, only one of them being transmitted to the newborn while the other persisting in the mother’s blood. Because of the increasing number of pregnant women coming from endemic areas for malaria, an accurate anamnesis of infant’s mother, and the inclusion of *Plasmodium* spp. research into TORCH screenings for mother-infant pair at birth, aiming at reducing morbidity and mortality associated to the disease might be suitable.

## Background

Congenital malaria is usually defined as the detection of asexual forms of *Plasmodium* spp. in a blood sample of a neonate during the first week of life or later if there is no possibility of postpartum infection by a mosquito bite (out of malaria endemic area) [1]. Congenital malaria can be acquired by transmission of parasites from the mother to child during pregnancy or perinatally during labour [2]. Congenital malaria in endemic countries is considered a rare condition due to the protective factors as the protection supplied by the placenta, the passive transfer of maternal antibodies [3] and the protective effect of fetal haemoglobin [4, 5]. The incidence of congenital malaria is highly variable. The literature reported an incidence between 7 and 33% in endemic area [6, 7] with an apparent increasing rate during the last years as result of rising drug resistance, increasing virulence of the parasite, human immunodeficiency virus (HIV) infection [7, 8]. The high variability seems related to several factors such as the different diagnostic methods and sampling (cord blood vs peripheral blood) used to detect *Plasmodium* spp., and the area in which the epidemiologic analyses are performed [6, 9]. In non-endemic countries, cases of congenital malaria are rare: in Europe only one case of congenital *Plasmodium falciparum* malaria was reported in 2014 [10]; in the USA only 81 cases of congenital malaria were identified between the years 1966 and 2005 [11]. Hereby, the case of a congenital malaria in an HIV-exposed child is reported.

## Case presentation

A 2-month-old male child was admitted to the Academic Department of Pediatrics of the Bambino Gesù Children’s Hospital (BGCH) due to anaemia and exposure to HIV. He was born prematurely in Italy by cesarean section at 34 weeks’ gestation after a bicorial, biamniotic pregnancy with birth weight of 2.080 kg. He was the first of non-identical twins. The mother was a 30-year-old migrant woman from Nigeria, who arrived in Italy at 27 weeks gestation. At presentation, she tested seropositive for HIV and cytomegalovirus (CMV) and started antiretroviral therapy. Her absolute lymphocyte count was 1410/µl; CD4 count and the HIV viral load were not reported in the documentation received from the Hospital where the mother was admitted in emergency when she arrived in Italy.

The twins were tested for HIV at birth with PCR for HIV-RNA searching. The female twin was positive for HIV and CMV infection, while the male twin was HIV negative at birth and treated with zidovudin as post-exposure prophylaxis for 6 weeks. TORCH screening (toxoplasmosis, rubella, cytomegalovirus, herpes simplex), abdominal and cerebral ultrasounds were performed to exclude other congenital infections on both twins. A week before admission at our Department the male twin was admitted to another hospital due to anaemia (Hb 5.1 g/dl), hence receiving a blood transfusion. On initial evaluation at BGCH, he was in good general condition, weighed 3.910 kg, with temperature of 36.5 °C, heart rate of 135 beats per minute, respiratory rate of 35 for minute. His abdomen was soft, the liver was palpable 4 cm below the right costal margin. The findings of the rest of the examination were unremarkable.

Laboratory tests at the admission, after a week from the first blood transfusion, revealed a leukocyte count of 12.000/mm^3^; a haemoglobin (Hb) level of 9.1 g/dl; a platelet count of 198.000/mm^3^ and a reticulocyte count of 169.000/mm^3^. His bilirubin level was 1.31 g/dl with direct bilirubin of 0.64 mg/dl; lactate dehydrogenase level of 945 UI/L and normal renal and liver function values.

A myelosuppression effect due to the zidovudin was initially hypothesized, then the haemoglobin concentration was monitored and a supportive therapy with folic acid and iron *per os* was started.

During hospitalization, a progressive decrease of Hb levels to 6.8 g/dl was observed, therefore, requiring additional blood transfusions. Causes of haemolytic anaemia and blood loss were excluded, due to persistently high reticulocyte count; also, direct and indirect Coombs and faecal occult blood tests were performed, resulting all of which were negative. Haemoglobin electrophoresis was also performed, although in the presence of blood transfusions, to exclude hereditary haemoglobinopathies. A subsequent physical examination was then performed, revealing an increase of spleen enlargement, also confirmed by ultrasound examination. A diagnostic of malaria was then considered.

Because of the infants’ age and the origin of the mother who came from an endemic area for malaria, the malaria panel provided in BGCH was performed on twins and mother’s blood. The panel included the following routine algorithm: (i) Rapid diagnostic test (RDT); (ii) microscopy of Giemsa-stained thick and thin blood smears for *Plasmodium* spp. identification (ID) and parasitaemia index assigned by two independent microscopists; (iii) molecular screening and typing of *Plasmodium* spp. by an end-point multiplex qualitative polymerase chain reaction (PCR) assay.

The RDT, based on either *Plasmodium* spp. lactate dehydrogenase (pLDH) and *P. falciparum* histidine-rich protein 2 (HRP2) antigens, was performed by using SD Bioline Malaria Antigen P.f/Pan (Standard Diagnostic), whose performance is periodically monitored by the World Health Organization Malaria Control Programmes [12].

Briefly, about PCR assay, DNA was extracted from 200 μl of EDTA blood with the QIAamp DNA Mini Kit (QIAGEN) and 5 μl of each DNA sample were probed with the 18S rRNA gene target of the multiplex PCR STAT-NAT Malaria Screening and Typing (Sentinel-Diagnostics). PCR products were visualized using 2.2% agarose (Lonza FlashGel^®^System) and a UV trans-illuminator BioRad.

**Fig. 1:**
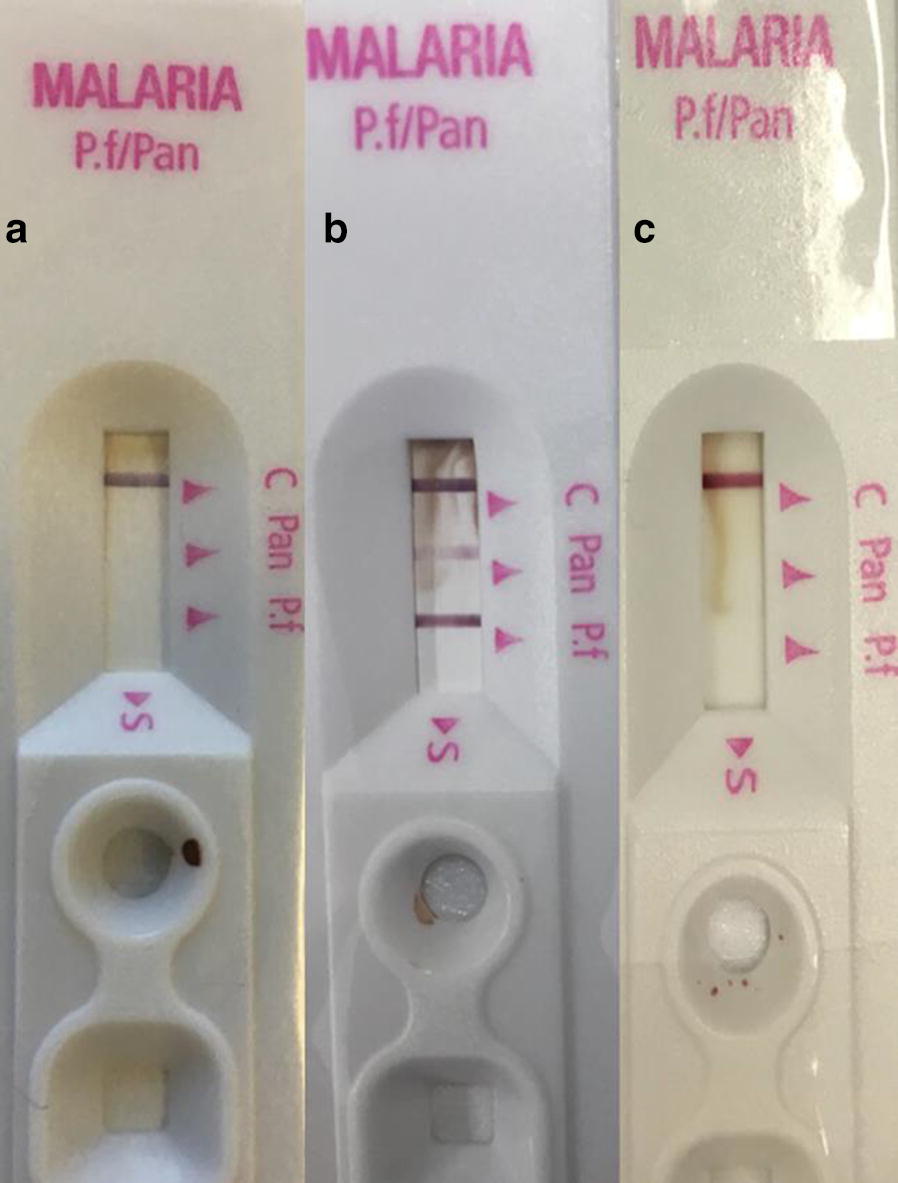
Pattern of RDT for the mother (**a**), the male twin (**b**), the female twin (**c**)

**Fig. 2:**
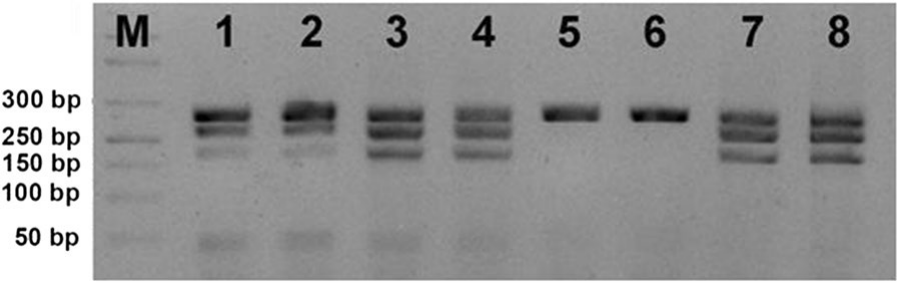
*Plasmodium* spp. screening by 18S rRNA targeting PCR. M DNA marker, (1/2) Mother’s sample replicates; (3/4) male infant’s sample replicates; (5/6) female infant’s sample duplicates; (7/8) male infant’s sample duplicates

**Fig. 3:**
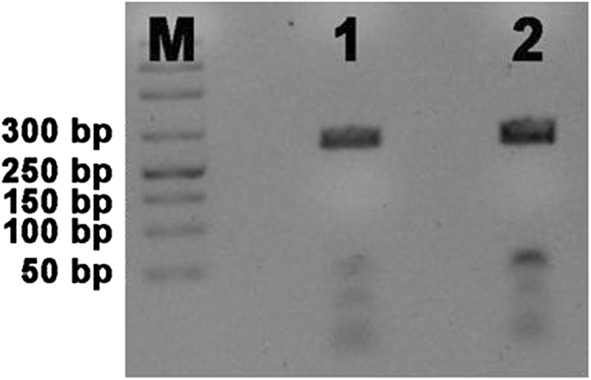
*Plasmodium falciparum* typing by 18S rRNA targeting PCR. M DNA marker, (1) Mother’s sample; (2) male infant’s sample

**Fig. 4:**
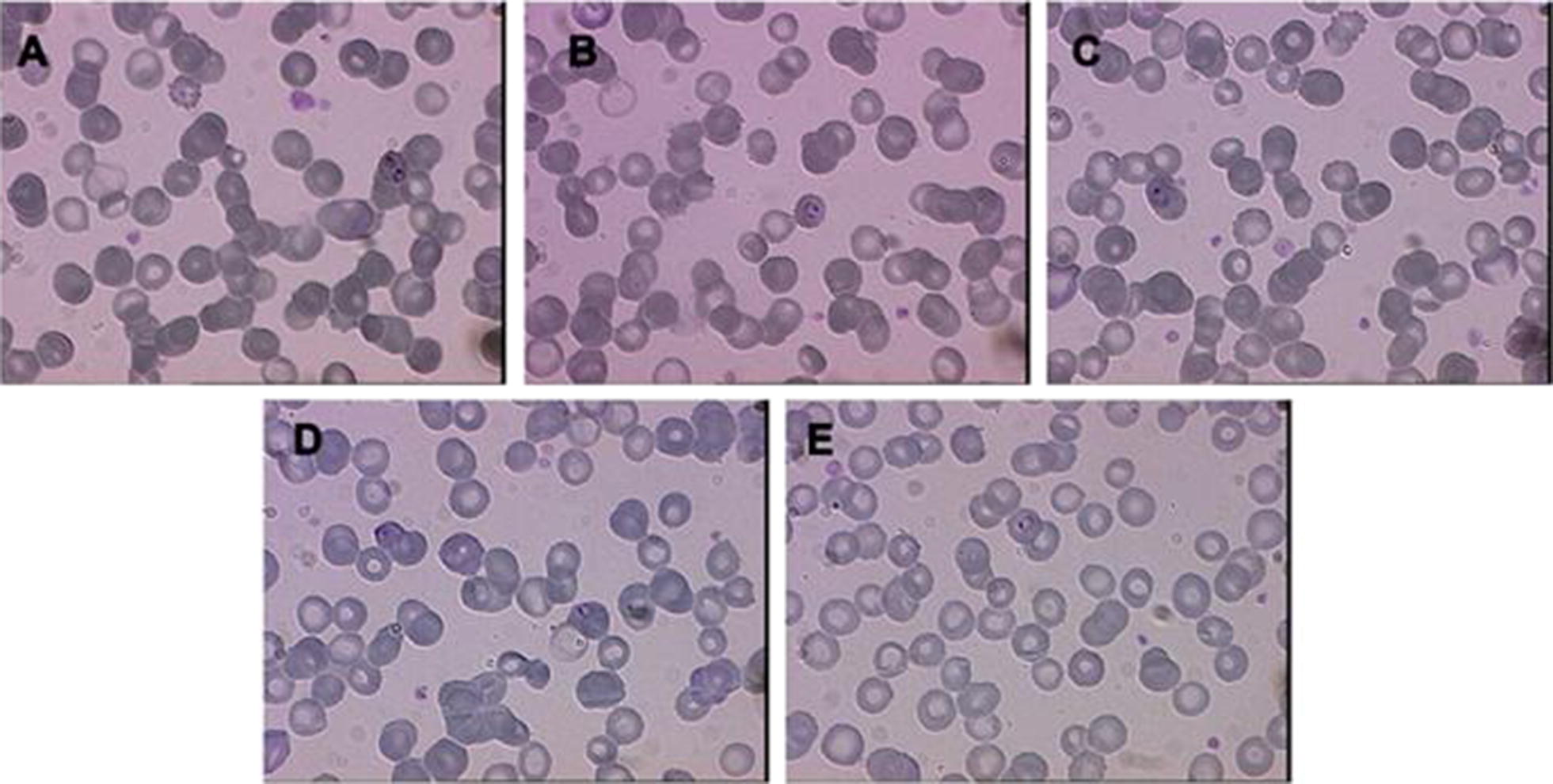
Infant and maternal blood smears. **A**–**C** Mother’s thin blood smear revealing *P. falciparum* immature trophozoites (ring forms) within erythrocytes. **D**, **E** Infant’s thin blood smear, obtained on the day of delivery, documenting the presence of *P. falciparum* trophozoites within erythrocytes

The RDT for *Plasmodium* spp. was negative for mother and female infant specimens, while male infant resulted positive (Fig. 1). PCR analysis confirmed a positive result for mother and male twin, revealing a *P. falciparum* infection, while samples from the other twin were consistently negative with both techniques (Figs. 2, 3). Thick and thin blood films stained by Giemsa revealed trophozoite forms of *P. falciparum* with parasitaemia index of 1% for the male infant and < 1% for the mother. The RBCs of the mother infected with malarial parasites were of normal size and poly-parasitized by trophozoites (Fig. 4).

Genotyping of *Plasmodium* spp. isolates was carried out to identify infectious clones in both mother and infant. The genotyping was performed by amplification of a neutral microsatellite marker (MS-TA109) [13] and four highly polymorphic markers: *P. falciparum* merozoite surface protein 1 (*Pfmsp1*) and its allelic subfamilies (K1, RO33, MAD20) [14], *P. falciparum* merozoite surface protein 2 (*Pfmsp2*) and its allelic subfamilies (3D7, FC27) [14], *P. falciparum* histidine-rich protein 2 (*Pfhrp2*) and t *P. falciparum* histidine-rich protein 3 (*Pfhrp3*) [15]. For allele detections, PCR was done in a 25 μl PCR mixture containing 10 μl of extracted DNA, 1× of MgCl_2_ free buffer Fast Start Roche, 2 mM of MgCl_2_, 200 μM of dNTPs, 10 μM of each primer and 0.25 U of FastStart Taq polymerase Roche. The cycling conditions for *Pfmsp1* were as follows: denaturation at 95 °C for 5 min, followed by 45 cycles at 94 °C for 30 min, annealing at 47 °C for 45 s and extension at 72 °C for 1.5 min and a final extension at 72 °C for 5 min. The cycling conditions for *Pfmsp1/Pfmsp2* families were: 95 °C for 5 min followed by 45 cycles at 94 °C for 1 min, 55 °C for 45 s, 72 °C for 1.5 min, and a final extension at 72 °C for 5 min. The *Pfhrp3* gene, FC27, K1 and TA109 microsatellites were amplified as described in Menegon et al. and Anderson et al. [13, 16]. The amplification products were analysed using a high-resolution capillary electrophoresis (QIAxcel Advanced system, Qiagen).

**Fig. 5:**
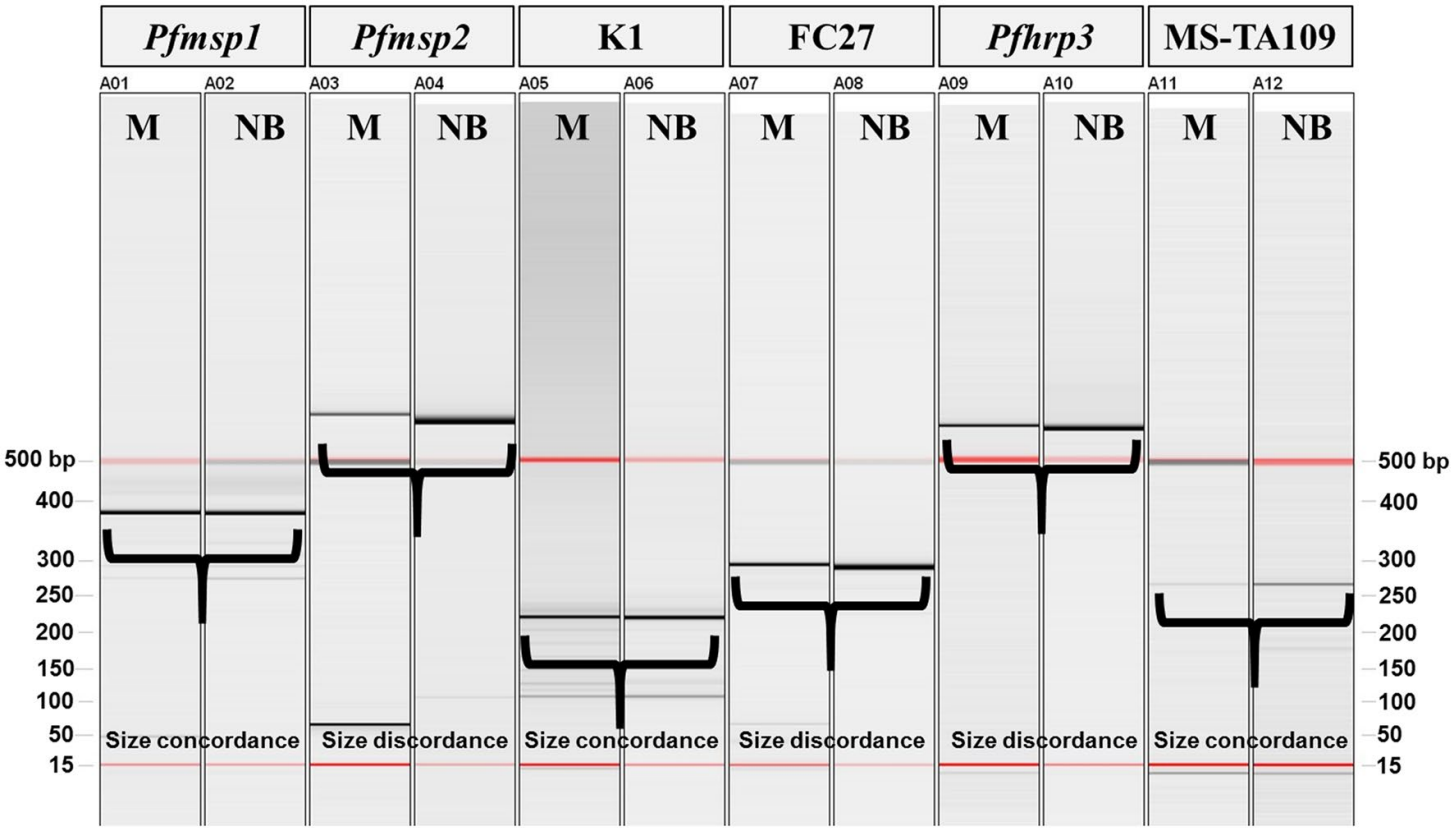
Electronic image of the gel displaying PCR product sizes of the six molecular markers amplified from mother and newborn DNA samples. The markers *Pfmsp2*, *FC27* (subfamily of *Pfmsp2*) and *Pfhrp3* showed discordant genotypes between the two analyzed samples

Genotypic characterization of *P. falciparum* isolates showed the presence of a single isolate in each of the analysed blood samples. All five *P. falciparum* polymorphic markers were genotyped for isolate present in the newborn’s infection, whereas only four markers (Ta109, *Pfmsp1*, *Pfmsp2* and *Pfhrp3*) were successfully amplified for the maternal isolate. Both isolates belonged to the K1 and the FC27 allelic subfamilies. The comparison of allelic profiles, based on length polymorphism of analysed markers, showed dissimilar size alleles for two molecular markers, *Pfmsp2* and *Pfhrp3*, indicating that two different parasite isolates were present in the mother and child at the time of blood collection, 2 month after delivery. Moreover, the amplification failure of *Pfhrp2* gene in the maternal sample was presumable due to the *hrp2*—deletion in the isolate infecting the mother (Fig. 5).

Because of the *P. falciparum* ID in the male infant, oral administration of atovaquone/proguanil (125 mg/50 mg daily for 3 days) was immediately started. The parasitaemia index on infant’s blood performed after treatment confirmed the clearance of the parasites; the following blood exams revealed a normalization of Hb level.

## Discussion and conclusions

This is the third case of congenital malaria ensued in a HIV-infected mother in a non-endemic country [11, 17]. A review of congenital malaria cases in non-endemic country, by referring to a period spanning the last 40 years was included. The database mined for data searching was PubMed and the keywords used were “congenital malaria cases” and “non-endemic countries”. The selected language was English. Congenital malaria is a rare disease in both non-endemic [10, 18] and endemic areas, the latter characterized by an incidence corresponding to 0.3–37% [19]. Among the 37 cases of congenital malaria in non-endemic country reported in the last 40 years, 21 (58%) were caused by *Plasmodium vivax*, including 1 in combination with *Plasmodium malariae* and 1 with *P. falciparum*; 8 by *P. falciparum* (22%); three cases caused by *P. malariae* and 2 by *Plasmodium ovale* (Table 1). Congenital malaria results from transplacental passage of parasites, which infect the infant in utero, or during delivery. Different mechanisms have been postulated: maternal transfusion into the fetal circulation, direct penetration of parasite through the chorionic villi or through premature separation of placenta [1]. Rarely, maternal history of malaria may not be reported and, therefore, it cannot be considered as a criterion for the diagnosis of congenital malaria [17]. Origin from endemic countries for malaria, fever during pregnancy, placental malaria and anaemia in the mother, are the main risk factors [1, 8].

**Table 1:** Congenital malaria cases reported in the last 40 years in non-endemic area [16]

Author/PMID	Endemic area	Interval time	Antenatal symptoms and treatment (if known)	Age at diagnosis	Symptoms at diagnosis	*Plasmodium* species and parasitemia	Haemoglobin level g/dl	Platelets count/µl	HIV status	Treatment	Country of diagnosis
Vernes et al. 1978 PMID: 353713	Cambodia	2 months	F 1 day after delivery	20 days	F	*P. vivax*	Normal	Normal	Unknown	Chloroquine	France
Excler et al. 1980 PMID: 6987619	Cambodia	1 year	F	1 day	F, liver and spleen enlargement	*P. vivax*	8	172,000	Unknown	Chloroquine	France
Lajarrige PMID: 18307546	Asia	Unknown	Delivery at home	12 days	F, paleness, LSE	*P. vivax*	3.8	159,000	Unknown	Chloroquine	France
Bour´ee et al. 1983 PMID:6340844	Cameroon	15 days	F	Birth	Lack of reactivity	*P. falciparum*	Unknown	85,000	Unknown	Unknown	France
Ch´eron et al. 1986 PMID:3813804	Guinea	7 months	Malaria during pregnancy	19 days	F	*P. ovale*	10	134,000	Unknown	Chloroquine	France
Peigne et al. 1987 PMID:3318635	Pakistan	4 days	Malaria 4 days after delivery	50 days	F, LSE, neurological	*P. vivax*	4.3	12,000	Unknown	Chloroquine	France
Poirrier PMID:18307546	Madagascar	17 months	F/chloroquine	19 days	F	*P. vivax* 1.3%	6.1	188,000	Unknown	Chloroquine	France
Ligny et al. 1989 PMID:2696411	Mali	18 days	Malaria during pregnancy	28 days	Paleness, LSE	*P. falciparum* 1.5%	5.2	Unknown	Unknown	Chloroquine	France
Hennequin et al. 1991 PMID:1819395	Cameroon	15 days	F	Birth	LSE, lethargy	*P. falciparum* 0.1%	Unknown	Unknown	Unknown	Chloroquine	France
Romand et al. 1994 PMID:8078837	Togo	14 months	F	60 days	F, paleness, LSE	*P. falciparum*	6.3	Unknown	Unknown	Halofantrine	France
Niyongabo et al. 1989 PMID:2654272	Laos	2 years	Quinine	19 days	F, haemolysis, irritability	*P. vivax* and *P. malariae*	10.8	60,000	Unknown	Quinine and chloroquine	France
Hindi and Azimi. 1980 PMID:7005857	Nigeria	1 year	Malaria during pregnancy	35 days	F, anaemia, LSE	*P. falciparum*	8.7	257,000	ng	Chloroquine	California
Park et al. 1984 PMID:12891034	Africa	0	Malaria during pregnancy	39 days	F, poor feeding, paleness, LSE	*P. vivax* 1%	12.6	45,000	ng	Chloroquine	Korea
Gouyon et al. 1986 PMID:3530172	Guyana	6 months	Malaria during pregnancy/4-aminoquinolein	21 days	F, poor feeding, paleness, LSE	*P. vivax* 1%	12.6	45,000	ng	Chloroquine	France
Lynk and Gold 1989 PMID:2594448	India (two cases)	6 months, 13 months	F during third trimester	28 days, 35 days	Irregular F, anorexia and lethargy, LSE, anaemia and thrombocytopeaenia	*P. vivax*	4.1 and 5.9	47,000	Unknown	Chloroquine	Canada
Joffe and Jadavji 1990 PMID:2196518	India (two cases)	9 months	Malaria (*P. vivax*) during pregnancy/chloroquine	21 days	F, diarrhoea, poor feeding, LSE, anaemia neutropenia and thrombocytopaenia	*P. vivax*	1	57,000	Unknown	Chloroquine	Canada
Subramanian et al. 1992 PMID:1520785	Salvador	4 months	F/antibiotics	15 days	F, coryza, anaemia	*P. vivax*	Unknown	52,000	Unknown	Chloroquine	Texas
Hulbert 1992 PMID:1576289	Guatemala	1 year	Asthenia	30 days	F, LSE, diarrhoea, anaemia	*P. vivax*	6.6	70,000	Unknown	Chloroquine	California
Alves 1995 PMID:14689015	Brazil	40 days	Unknown	14 days	Unknown	*P. vivax*	Unknown	Unknown	Unknown	Unknown	São Paulo State
Lee et al. 1996 PMID:9046213	Pakistan	Unknown	F/Ibuprofen	60 days	F, anaemia, haemolysis, cough, paleness, LSE	*P. vivax*	5.3	69,000	ng	Chloroquine	Singapore
Marques et al. 1996 PMID:14688962	Brazil (two cases)	Unknown	Malaria during pregnancy	Unknown	Anaemia, LSE	*P. vivax, P. falciparum*	Unknown	Unknown	Unknown	unknown	São Paulo state
Kuyucu et al.1999 PMID:10770683	Turkey	Unknown	F and chills/Chloroquine	19 days	F, poor feeding, haemolysis, anaemia, LSE	*P. vivax*	8.5	50,000	ng	Chloroquine	Turkey
Niederer and Loeffler 1999 PMID:9951993	India	1 year	Unknown	23 days	F, cough, irritability, poor feeding, anaemia, leucopaenia, thrombocytopaenia	*P. vivax*	10.7	27,000	Unknown	Chloroquine	California
Romero Urbano et al. 2000 PMID:11003930	Guinea	1 month	Unknown	21 days	F, anaemia, thrombocytopenia	*P. falciparum*	Unknown	Unknown	Unknown	Mefloquine	Spain
Zenz et al. 2000 PMID:10890139	Ghana	18 months	Unknown	56 days	F, LSE, anaemia	*P. falciparum* and *P. malariae*	8.3	Unknown	Unknown	Chloroquine	Germany
D’avanzo MMWR, March 1, 2002/51(08); 16-5	Congo	5 years	Malaria 5 years before pregnancy/chloroquine	21 days	F, dark urine, respiratory troubles, anaemia	*P. malariae*	6.6	109,000	ng	Chloroquine	North Carolina, USA
Olowu et al. 2002 PMID:12221966	Nigeria		Unknown	8 h	unknown	unknown	Unknown	Unknown	Unknown	Chloroquine	Osun State, Nigeria
Doraiswamy CDC-MMRW April 22, 2005/54(15);383–384	Guatemala	5 months	F, coryza	49 days	Moderate F, anaemia	*P. vivax*	6.2	Unknown	Unknown	Chloroquine	New York, USA
Siriez et al. 2005 PMID:16465819	Congo	2 years	Unknown	42 days	F, haemolysis, anaemia, thrombocytopaenia, poor feeding, LSE	*P. malariae* 3%	5.8	110,000	HIV-1	Chloroquine	France
Del Castillo et al. 2017 PMID:28077745	Nigeria	3 months	Pueperal F, thrombocytopenia during delivery	14 days	F, cough	*P. falciparum* 5.4%	Unknown	Unknown	Unknown	Quinidine and Clindamycin	Washington (Columbia)
Del Punta et al. 2010 PMID:20193072	Pakistan	1 year	F, anaemia, thrombocytopenia during delivery	22 days	F, paleness, whining cry, liver and spleen enlargement,	*P. vivax* 2%	12.3	14,000	Unknown	Chloroquine	Italy
Voittier et al. 2008 PMID:18307546	Guyana	6 months	malaria *P. vivax*/4-aminoquinoline	21 days	F, paleness, liver and spleen enlargement	*P. vivax* 1%	12.6	45,000	Unknown	Chloroquine	France
Voittier et al.2008 PMID:18307546	Angola	3 years	HIV	19 days	F, poor feeding	*P. ovale* 2%	12	38,000	ng	Chloroquine	France
Hagmann et al. 2007 PMID:17505278	Honduras	9 months	Malaria during pregnancy	26 days	F, cough, runny nose	*P. vivax* 4–5%	11.4	313,000	Unknown	Chloroquine	New York, USA
De Pontual et al. 2006 PMID:17030531	Congo	2 years	HIV	42 days	F, liver and spleen enlargement	*P. malariae* 1.8%	6.4	122,000	ng	Chloroquine	France
Hewson et al. 2003 PMID:14629507	India	4 months	F, abdominal pain, rigors	19 days	Apnoea, bradycardia	*P. vivax*	12.2	95,000	Unknown	Chloroquine	South Australia

*F* fever, *LSE* liver spleen enlargement, *ng* negative

HIV infection increases susceptibility to malaria during pregnancy [7] and it is associated with higher parasite density, higher risk of maternal and fetal anaemia, intra-uterine growth retardation (IUGR) and pre-term delivery [20], and low birth weight (LBW) in the neonates [21]. Recently a higher prevalence of congenital malaria in infants of mothers co-infected with HIV and malaria have been reported [8]. HIV infection compromised maternal immunity though an impairment of antibody responses with a higher risk of *P. falciparum* transmission [22]. However, the mechanism by which HIV increases susceptibility to malaria is not known. After birth, the mother may have a normal physical examination and negative blood malaria parasite [17]. In the present case, the mother never suffered from fever or symptoms suggestive for malaria during pregnancy. In women from endemic countries for malaria and with previous episodes of malaria it is common to be asymptomatic because of the immunity developed during the time [23].

In most cases of congenital malaria, the diagnosis is made at 10–28 days of age; 20 of the 37 published cases (56%) were diagnosed before 21 days (Table 1). The symptoms are rarely detected at birth, possibly because of the presence of IgG transferred from the mother during the pregnancy, and the protective effect of HbF; indeed, the passive immunity may prevent delay the onset of congenital malaria up to 6 weeks [24].

Clinical features of congenital malaria include fever, anaemia, thrombocytopaenia, liver and spleen enlargement. Jaundice, regurgitation, loose stools and poor feeding, occasionally apnea and cyanosis have also been reported [1]. Such clinical features may be confused with bacterial or viral infection, leading to a delay in diagnosis [25]. The patient presented anaemia with high level of reticulocyte. He needed a blood transfusion every week, a progressive spleen enlargement was noted. Initially toxicity by antiretroviral therapy was hypothesized because of the good clinical condition and the absence of symptoms and signs suggestive for infection. No fever was detectable during the entire hospitalization. Five other cases of congenital malaria without fever have been described (Table 1). In this case, there was not record of the exact onset of anaemia because blood tests were not performed during the period 7–30 days after birth. Likely, the anaemia occurred days and even weeks before admission to hospital.

The mother of the infant travelled during pregnancy in region where a high percentage of *P. falciparum* chloroquine resistance is reported, finally arriving to a non-endemic country [26, 27]. Because of the stable clinical condition and for the suspect of chloroquine resistance, the authors decided to treat the infant, with atovaquone/proguanil according to CDC guidelines [28]. Because the parasitaemia index was 1% and no criteria of severe malaria were present, oral administration was considered as appropriate treatment. *Plasmodium* spp. on female twin’s blood was absent, as reported for other cases in the literature [29–36]. Peripheral maternal and peripheral newborn’s parasite populations was analysed 2 month after delivery to compare allelic profile of persistent *P. falciparum* isolates. Two different parasite isolates in the mother and child at the time of blood collection were found. Likely during the gestation the mother was parasitized by different *P. falciparum* strains, as supported by literature data [37]. It is conceivable for the mother to have harboured more than one isolate during pregnancy, only one of them being transmitted to the newborn and the different one having persisted in the mother’s blood after delivery [38].

Malaria RDTs are useful tools to confirm presence of malaria. Under optimal conditions, the sensitivity of the RDTs is considered similar to that of direct microscopy [39]. However, their execution may be questionable since on a number of occasions false negative results have been encountered which would negatively affect proper early therapeutic intervention. The three main groups of antigens detected by RDTs are HRP2, produced by trophozoites and young gametocytes of *P. falciparum* only; pLDH enzyme (*P. falciparum* specific, *P. vivax* specific or pan specific), and aldolase pan-specific enzyme [40].

In the presented case, RDTs of peripheral blood failed to detect a maternal infection, while PCR and microscopy were highly effective. PfHRP2 is a histidine and alanine-rich protein, characterized by a highly polymorphic repeat domain and represents the most common malaria antigen targeted by RDTs for the specific diagnosis of *P. falciparum* [41]. Frequently, another protein of *P. falciparum*, the PfHRP3 antigen [42], is recognized by PfHRP2-based RDTs [40, 43]. The above studies further revealed that polymorphisms of the *Pfhrp*2/3 genes can affect the performance of HRP2-based RDTs in term of sensitivity up to total test failure (false-negative), recommending molecular investigation. False negatives can be also due to impairment in host and parasite density, or antigen concentration.

Finally, *P. falciparum* parasites not expressing PfhHRP2 and/or PfHRP3 antigens have been reported [44]. These results are consistent with those reported in the literature [45–49], and suggest that diagnostic guidelines for malaria be revisited.

The negative RDT in the mother can be justified by a low parasitaemia index and by the possible deletion of the *Pfhrp*2 gene in this parasite. As in the present case, the discordance in vertical transmission of malaria in bicorial and biamniotic pregnancy is reported in the literature [29], as well for CMV, HIV and toxoplasmosis [32, 33, 35]. Therefore, the same pathogenesis was supposed for the present case.

## Conclusion

**Fig. 6:**
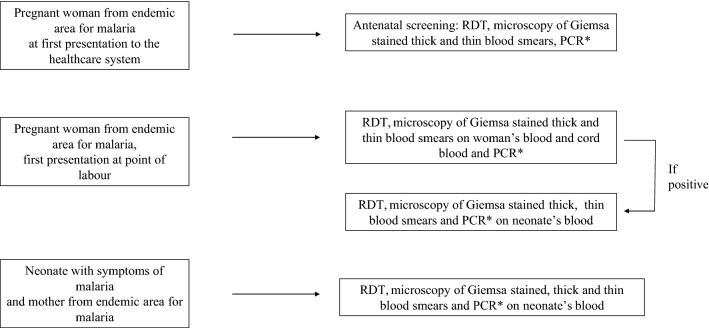
Hypothesis of diagnostic algorithm for congenital malaria. Asterisk If PCR for *Plasmodium* spp is available in the Hospital

A prompt diagnosis of congenital malaria is crucial. The increasing number of pregnant women travelling from endemic areas for malaria to non-endemic countries, calls for routine investigation of *Plasmodium* spp. in women and neonates at risk: (i) women and pregnant women from endemic area for malaria, (ii) all neonates and infants with fever, anaemia, thrombocytopaenia and hepatosplenomegaly with mother who have travelled or lived in non-endemic area for malaria (Fig. 6).

In such cases, accurate anamnesis of neonate’s mother and inclusion of *Plasmodium* spp. search into the TORCH screening for mother and infant at birth should be performed, to avoid delay in the diagnosis and to reduce morbidity and mortality associated to the disease. The differential diagnosis between neonatal malaria vs neonatal sepsis is not easily to be resolved by the use of clinical features alone. However, also the laboratory diagnosis of low parasitaemia, such as that observed in mother-infant pair infections, require high level of expertise in malaria diagnostic panels. Use of malaria RDT assays that can detect antigens other than PfHRP2 and pLDH should could be strongly encouraged in field setting but also in hospitals, in order to enhance malaria diagnosis. Advanced malaria diagnostic panels, when possible, can be decisive to monitor both congenital and other malaria infections during perinatal and paediatric ages.

## Abbreviations

HIV: human immunodeficiency virus
TORCH: toxoplasmosis, rubella, cytomegalovirus, herpes simplex
RDT: rapid diagnostic test
LDH: lactate dehydrogenase
hrp2: histidine-rich protein 2
MS-TA109: microsatellite-(TA)n
*Pfmsp1*: *Plasmodium falciparum* merozoite surface protein 1
*Pfmsp2*: *Plasmodium falciparum* merozoite surface protein 2
*Pfhrp3*: *Plasmodium falciparum* histidine-rich protein 3
LBW: low birth weight
IUGR: intra-uterine growth retardation
WHO: World Health Organization
